# Supplementary material for: Cite-seeing and reviewing: A study on citation bias in peer review
Source: PLoS One. 2023 Jul 7;18(7):e0283980. doi: 10.1371/journal.pone.0283980 (PMC10328240; doi:10.1371/journal.pone.0283980)
Supplement: S1 Appendix — (PDF) [file pone.0283980.s001.pdf]

Appendix

In the appendix we provide additional details on our analysis procedure.

A Controlling for confounding factors

As described in Section 3.2.2, our analysis relies on a number of characteristics (`quality`, `expertise`, `preference`, `seniority`) to account for confounding factors C2–C5. The value of `quality` is, of course, unknown and we exclude it from the analysis by focusing on differences in reviewers' evaluations made for the same submission (details in Appendix 5). For the remaining characteristics, we use a number of auxiliary variables available to conference organizers to quantify these characteristics. These variables differ between conferences and Table 3 summarizes the details for both venues.

Table 3. Description of variables used in the analysis.

Characteristic	Auxiliary variable	EC 2021	ICML 2020
expertise	Self-reported expertise	In both venues, reviewers were asked to self-evaluate their ex post expertise in reviewing submissions using a 4-point Likert item. The evaluations were submitted together with initial reviews and higher values represent higher expertise. We encode these evaluations in a continuous variable `expertiseSRExp`.	
	Self-reported confidence	Not used in the conference.	Similar to expertise, reviewers were asked to evaluate their ex post confidence in their evaluation on a 4-point Likert item. We encode these evaluations in a continuous variable `expertiseSRConf`.
	Textual overlap	Not used in the conference.	TPMS measure of textual overlap [22] between a submission and a reviewer's past papers (real value between 0 and 1; higher values represent higher overlap). We denote this quantity `expertiseText`. Out of 3,335 (submission, reviewer) pairs that qualify for the analysis (before data filtering is executed), 439 pairs have the value of `expertiseText` missing due to reviewers not creating their TPMS accounts. Entries with missing values were removed from the analysis.
preference	Self-reported preference	Reviewers reported partial rankings of submissions in terms of their preference in reviewing them by assigning each submission a non-zero value from -100 to 100 (the higher the value the higher the preference; non-reported preferences are encoded as 0). In the automated assignment, reviewers were not assigned to papers with negative preferences. Assignment of submissions to reviewers who did not enter a preference was discouraged, but not forbidden. For analysis, we transform non-negative preferences into percentiles `prefPerc` (0 means top preference, 100 – bottom).	Reviewers bid on submissions by reporting a value from 2 (Not willing to review) to 5 (Eager to review). In the automated assignment, reviewers were not assigned to papers with bids of value 2. Assignment of submissions to reviewers who did not enter a bid was discouraged, but not forbidden. As a result, out of 3,335 (submission, reviewer) pairs that qualify for the analysis (before data filtering is executed), 159 pairs had the value of bid missing. Entries with missing values were removed from the analysis. Positive bids (3, 4, 5) are captured in the continuous variable `prefBid`.
	Missing preference	Out of 849 (submission, reviewer) pairs that qualify for the analysis (before data filtering is executed), 154 have the reviewer's preference missing. This missingness is captured in a binary indicator `missingPref`.	Not used in the analysis as data points with missing preferences are excluded from the analysis.
seniority	Manual classification	Program chairs split the reviewer pool in two groups: *curated* — reviewers with significant review experience or personally recommended by senior members of the program committee; *self-nominated* — reviewers who nominated themselves and satisfied mild qualification requirements.	Program chairs split the reviewer pool in two groups: *senior* and *junior*.
		In both venues, the split into groups was encoded in a binary variable `seniority` that equals 1 when a reviewer was assigned to *curated* or *senior* group and 0 otherwise.	
