# Supplementary material for: Cite-seeing and reviewing: A study on citation bias in peer review
Source: PLoS One. 2023 Jul 7;18(7):e0283980. doi: 10.1371/journal.pone.0283980 (PMC10328240; doi:10.1371/journal.pone.0283980)
Supplement: S2 Appendix — (PDF) [file pone.0283980.s002.pdf]

# B Details of the parametric inference

Conceptually, the parametric analysis of both EC 2021 and ICML 2020 data is similar up to the specific implementation of our model (1) in these venues. In this section, we specify this parametric model for both venues using variables introduced in Table 3, and introduce the procedure used to eliminate the `quality` variable whose values are unobserved.

## Specification of parametric model

We begin by specifying the model (1) to each of the venues we consider in the analysis.

**ICML** With auxiliary variables introduced in Table 3, our model (1) for ICML reduces to the following specification:

$$\begin{aligned}\texttt{score} \sim \alpha_0 + \alpha_1 \cdot \texttt{quality} + \alpha_2^{(1)} \cdot \texttt{expertiseSRExp} + \alpha_2^{(2)} \cdot \texttt{expertiseSRConf} \\ + \alpha_2^{(3)} \cdot \texttt{expertiseText} + \alpha_3 \cdot \texttt{prefBid} + \alpha_4 \cdot \texttt{seniority} + \alpha^* \cdot \texttt{citation}.\end{aligned}$$

**EC** An important difference between our ICML and EC analyses is that in the latter we do not remove entries with missing values of auxiliary variables but instead incorporate the data missingness in the model. For this, recall that in EC, the only source of missingness is reviewers not reporting their `preference` in reviewing submissions. To incorporate this missingness, we enhance the model by an auxiliary binary variable `missingPref` that equals one when the `preference` is missing and enables the model to accommodate associated dynamics:

$$\begin{aligned}\texttt{score} \sim \alpha_0 + \alpha_1 \cdot \texttt{quality} + \alpha_2 \cdot \texttt{expertiseSRExp} + \alpha_3^{(1)} \cdot \texttt{prefPerc} \\ + \alpha_3^{(2)} \cdot \texttt{missingPref} + \alpha_4 \cdot \texttt{seniority} + \alpha^* \cdot \texttt{citation}.\end{aligned}$$

## Elimination of submission quality from the model

Having the conference-specific models defined, we now execute the following procedure to exclude the unobserved variable `quality` from the analysis. For ease of exposition, we illustrate the procedure on the model (1) as details of this procedure do not differ between conferences.

**Step 1. Averaging scores of CITED and UNCITED reviewers** Each submission used in the analysis is assigned to at least one CITED and at least one UNCITED reviewer. Given that there may be more than one reviewer in each category, we begin by averaging the scores given by CITED and UNCITED reviewers to each submission. The linear model assumptions behind our model (1) ensure that for each submission, averaged scores $\texttt{score}_{\text{ctd}}$ and $\texttt{score}_{\text{unctd}}$ also adhere to the following linear models:

$$\begin{aligned}\texttt{score}_{\text{ctd}} \sim & \alpha_0 + \alpha_1 \cdot \texttt{quality} + \alpha_2 \cdot \texttt{expertise}_{\text{ctd}} + \alpha_3 \cdot \texttt{preference}_{\text{ctd}} \\ & + \alpha_4 \cdot \texttt{seniority}_{\text{ctd}} + \alpha^*,\end{aligned} \tag{2}$$

$$\begin{aligned}\texttt{score}_{\text{unctd}} \sim & \alpha_0 + \alpha_1 \cdot \texttt{quality} + \alpha_2 \cdot \texttt{expertise}_{\text{unctd}} + \alpha_3 \cdot \texttt{preference}_{\text{unctd}} \\ & + \alpha_4 \cdot \texttt{seniority}_{\text{unctd}}.\end{aligned} \tag{3}$$

In these equations, subscripts "ctd" and "unctd" represent means of the corresponding values taken over CITED and UNCITED reviewers, respectively. Variances of the corresponding Gaussian noise in these models are inversely proportional to the number of CITED reviewers (2) and the number of UNCITED reviewers (3).

**Step 2. Taking difference between mean scores** Next, for each submission, we take the difference between mean scores $\texttt{score}_{\text{ctd}}$ and $\texttt{score}_{\text{unctd}}$ and observe that the linear model assumptions again ensure that the difference ($\texttt{score}_{\Delta}$) also follows the linear model:

$$\texttt{score}_{\Delta} \sim \alpha_2 \cdot \texttt{expertise}_{\Delta} + \alpha_3 \cdot \texttt{preference}_{\Delta} + \alpha_4 \cdot \texttt{seniority}_{\Delta} + \alpha^*. \quad (4)$$

Subscript $\Delta$ in this equation denotes the difference between the mean values of the corresponding quantity across CITED and UNCITED conditions: $X_{\Delta} = X_{\text{ctd}} - X_{\text{unctd}}$. Observe that by taking a difference we exclude the original intercept $\alpha_0$ and the unobserved quality variable from the model. Thus, all the variables in the resulting model (4) are known and we can fit the data we have into the model. Each submission used in the analysis contributes one data point that follows the model (4) with a submission-specific level of noise:

$$\sigma^2 = \sigma_0^2 \left( {}^1\!/\!\#\textsc{cited} + {}^1\!/\!\#\textsc{uncited} \right),$$

where $\sigma_0^2$ is the level of noise in the model (1) that defines individual behavior of each reviewer.

**Step 3. Fitting the data** Having removed the unobserved variable quality from the model, we use the weighted linear regression algorithm implemented in the R stats package to test for significance of the target coefficient $\alpha^*$.
