# Supplementary material for: Cite-seeing and reviewing: A study on citation bias in peer review
Source: PLoS One. 2023 Jul 7;18(7):e0283980. doi: 10.1371/journal.pone.0283980 (PMC10328240; doi:10.1371/journal.pone.0283980)
Supplement: S3 Appendix — (PDF) [file pone.0283980.s003.pdf]

# C Details of the non-parametric inference

Non-parametric analysis conducted in ICML 2020 consists of two steps that we now discuss.

**Step 1. Matching** First, we conduct matching of (submission, reviewer) pairs by executing the following procedure separately for each submission. Working with a given submission $\mathcal{S}$, we consider two groups of reviewers assigned to $\mathcal{S}$: CITED and UNCITED. Next, we attempt to find CITED reviewer $\mathcal{R}_{\text{ctd}}$ and UNCITED reviewer $\mathcal{R}_{\text{unctd}}$ that are similar in terms of `expertise`, `preference`, and `seniority` characteristics. More formally, in terms of variables we introduced in Table 3, reviewers $\mathcal{R}_{\text{ctd}}$ and $\mathcal{R}_{\text{unctd}}$ should satisfy *all of the following criteria* with respect to $\mathcal{S}$:

- Self-reported expertise of reviewers in reviewing submission $\mathcal{S}$ is the same:

$$\texttt{expertiseSRExp}_{\text{ctd}} = \texttt{expertiseSRExp}_{\text{unctd}}$$

- Self-reported confidence of reviewers in their evaluation of submission $\mathcal{S}$ is the same:

$$\texttt{expertiseSRConf}_{\text{ctd}} = \texttt{expertiseSRConf}_{\text{unctd}}$$

- Textual overlap between submission $\mathcal{S}$ and papers of each of the reviewers differ by at most 0.1:

$$|\texttt{expertiseText}_{\text{ctd}} - \texttt{expertiseText}_{\text{unctd}}| \leq 0.1$$

- Reviewers' bids on sibmission $\mathcal{S}$ satisfy one of the two conditions:
  1. Both bids have value 3 ("In a pinch"):

  $$\texttt{prefBid}_{\text{ctd}} = \texttt{prefBid}_{\text{unctd}} = 3$$

  2. Both bids have values greater than 3 (4-"Willing" or 5-"Eager"):

  $$\texttt{prefBid}_{\text{ctd}} \in \{4, 5\} \quad \text{and} \quad \texttt{prefBid}_{\text{unctd}} \in \{4, 5\}$$

- Reviewers belong to the same seniority group:

$$\texttt{seniority}_{\text{ctd}} = \texttt{seniority}_{\text{unctd}}$$

We run this procedure for all submissions in the pool. If for submission $\mathcal{S}$ there are no reviewers $\mathcal{R}_{\text{ctd}}$ and $\mathcal{R}_{\text{unctd}}$ that satisfy these criteria, we remove submission $\mathcal{S}$ from the non-parametric analysis. Overall, we let $K$ denote the number of such 1-1 matched pairs obtained and introduce the set of triples that the remaining analysis operates with:

$$\left\{ \left[ (\mathcal{S}^{(i)}, \mathcal{R}^{(i)}_{\text{ctd}}, \mathcal{R}^{(i)}_{\text{unctd}}) \right] \right\}_{i=1}^{K}. \tag{5}$$

Each triple in this set consists of submission $\mathcal{S}$ and two reviewers $\mathcal{R}_{\text{ctd}}$ and $\mathcal{R}_{\text{unctd}}$ that (i) are assigned to $\mathcal{S}$ and (ii) satisfy the aforementioned conditions with respect to $\mathcal{S}$. Within each submission, each reviewer can be a part of only one triple.

Let us now consider two (submission, reviewer) pairs associated with a given triple. Observe that these pairs share the submission, thereby sharing the value of unobserved characteristic `quality`. Additionally, the criteria used to select reviewers $\mathcal{R}_{\text{ctd}}$ and $\mathcal{R}_{\text{unctd}}$ ensures that characteristics `expertise`, `preference`, and `seniority` are also similar across these pairs. Crucially, while being equal on all four characteristics, these pairs have different values of the `citation` indicator.

**Step 2. Permutation test** Having constructed the set of triples (5), we now compare scores given by CITED and UNCITED reviewers within these triples. Specifically, consider triple $i \in \{1, \ldots, K\}$ and let $Y_{\text{ctd}}^{(i)}$ (respectively, $Y_{\text{unctd}}^{(i)}$) be the score given by CITED reviewer $\mathcal{R}_{\text{ctd}}^{(i)}$ (respectively, UNCITED reviewer $\mathcal{R}_{\text{unctd}}^{(i)}$) to submission $\mathcal{S}^{(i)}$. Then the test statistic $\tau$ of our analysis is defined as follows:

$$\tau = \frac{1}{K} \sum_{i=1}^{K} \left( Y_{\text{ctd}}^{(i)} - Y_{\text{unctd}}^{(i)} \right). \tag{6}$$

To quantify the significance of the difference between scores given by CITED and UNCITED reviewers, we execute the permutation test. Specifically, at each of the 10,000 iterations, we independently permute the `citation` indicator within each triple $i \in \{1, \ldots, K\}$. For each permuted sample, we recompute the value of the test statistic (6) and finally check whether the actual value of the test statistic $\tau$ appears to bee "too extreme" for the significance level 0.05.
