# Supplementary material for: Cite-seeing and reviewing: A study on citation bias in peer review
Source: PLoS One. 2023 Jul 7;18(7):e0283980. doi: 10.1371/journal.pone.0283980 (PMC10328240; doi:10.1371/journal.pone.0283980)
Supplement: S4 Appendix — (PDF) [file pone.0283980.s004.pdf]

D Model diagnostics

Conclusions of our parametric analysis depend on the linear regression assumptions that we cannot a priori verify. To get some insight on whether these assumptions are satisfied, we conduct basic model diagnostics. Visualizations of these diagnostics are given in Figure 2 (EC 2021) and Figure 3 (ICML 2020). Overall, the diagnostics we conduct do not reveal any critical violations of the underlying modeling assumptions and suggest that our linear model (1) provides a reasonable fit to the data.

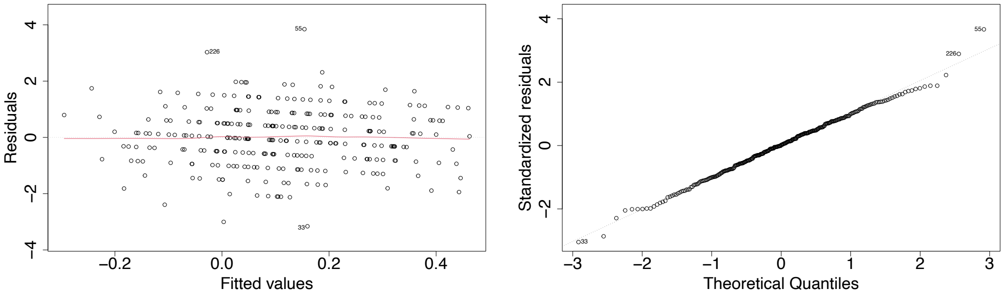

(a) Residuals vs Fitted **(b)** Normal Q-Q

Fig 2. Model diagnostics for the EC 2021 parametric analysis. Residuals do not suggest any critical violation of model assumptions.

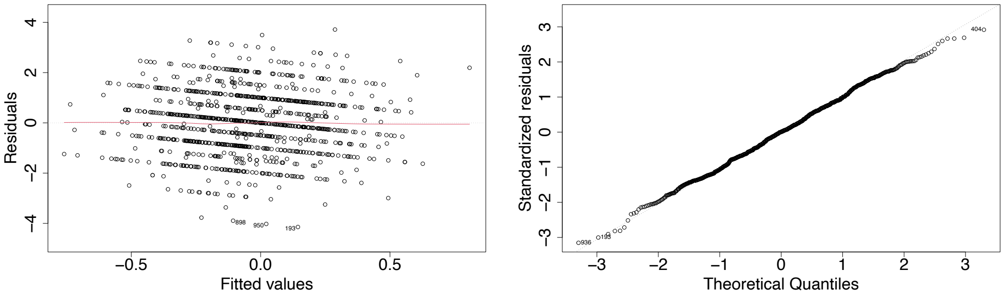

(a) Residuals vs Fitted **(b)** Normal Q-Q

Fig 3. Model diagnostics for the ICML 2020 parametric analysis. Residuals do not suggest any critical violation of model assumptions.
