# Supplementary material for: Cite-seeing and reviewing: A study on citation bias in peer review
Source: PLoS One. 2023 Jul 7;18(7):e0283980. doi: 10.1371/journal.pone.0283980 (PMC10328240; doi:10.1371/journal.pone.0283980)
Supplement: S1 File — (PDF) [file pone.0283980.s005.pdf]

Comparison of Review Scores of Computer Science Conference Submissions With Cited and Uncited Reviewers

Objective: Many anecdotes suggest that including citations to the works of potential reviewers is a good (albeit unethical) way to increase the acceptance chances of a paper. However, previous attempts[1,2] to quantify this effect had low sample size and unaccounted confounding factors such as paper quality (stronger papers have longer bibliographies) or reviewer expertise (cited reviewers have higher expertise). In this work, the question of whether reviewers are positively influenced when their work is cited in the paper they review was investigated.

Design: The study used data from two top-tier computer science conferences—2021 Association for Computing Machinery Conference on Economics and Computation (EC) and 2020 International Conference on Machine Learning (ICML). Both conferences receive full papers that undergo rigorous review (similar to top journals in other areas). The study analyzed anonymized observational data and consent collection was not required.

The dependent variable of the analysis was the overall score given by a reviewer to a submission (between 1-5 in EC and between 1-6 in ICML; higher means better). To investigate the relationship between the citation of a reviewer and their score, parametric (linear regression for EC and ICML) and non-parametric (permutation test with 1-1 covariate matching for ICML) tests were combined, circumventing various confounding factors: paper quality, genuinely missing citations, reviewer expertise, reviewer seniority, and reviewers' preferences in which submissions to review.

The approach consisted of matching cited and uncited reviewers within each paper and then carefully analyzing the differences in their scores. In this way, the aforementioned "paper quality" confounder was alleviated as matched cited and uncited reviewers reviewed the same paper. Additionally, various attributes of reviewers (e.g., their expertise in the paper's research area) were used to account for confounders associated with the reviewer identity (e.g., reviewer expertise). Finally, the "genuinely missing citation" confounder was accounted for by excluding papers in which an uncited reviewer genuinely decreased their evaluation of a paper because it failed to cite their own relevant past work.

Results: Overall, three analyses were conducted, with sample sizes ranging from 60 to 1031 papers and from 120 to 2757 reviewers' evaluations. These analyses detected citation bias in both venues and indicated that citation of a reviewer is associated with an increase in their score (approximately 0.23 on a 5-point Likert item). For reference, a one-point increase of a score by a single reviewer improves the position of a submission by 11% on average.

Conclusions: To improve peer review, it is important to understand the biases present and their magnitude. This work[3] studied citation bias and raised an important open problem of mitigating the bias. The reader should be aware of the observational nature of this study when interpreting the results.

1. Beverly R, Allman M. Findings and Implications from Data Mining the IMC Review Process. ACM SIGCOMM Computer Communication Review. January 9, 2012;43(1):22-29. doi:10.1145/2427036.2427040

2. Sugimoto CR, Cronin B. Citations gamesmanship: testing for evidence of ego bias in peer review. Scientometrics. June 1, 2013;95(3):851-862. doi: 10.1007/s11192-012-0845-z

3. Stelmakh I, Rastogi C, Liu R, Echenique F, Chawla S, Shah NB. Cite-seeing and Reviewing: A Study on Citation Bias in Peer Review, 2022, arXiv preprint arXiv:2203.17239

	**Analysis 1**	**Analysis 2**	**Analysis 3**
Conference	EC 2021	ICML 2020	ICML 2020
Analysis Method	Regression	Regression	1-1 matching on covariates
Sample Size (# papers, # reviewers)	283, 152	1031, 1565	60, 115
Sample Size (# evaluations by cited, uncited reviewer)	427, 413	1140, 1617	60, 60
Effect Size (Increase in a score associated with citation bias), confidence intervals, and P values	0.23 on 5-point scale [0.06, 0.40]; P=0.009	0.16 on 6-point scale [0.05, 0.27]; P=0.004	0.42 on 6-point scale [0.10, 0.73]; P=0.02

Table 1. Results of the experiment. Data suggests that citation bias is present both in EC 2021 and ICML 2020. Confidence intervals for A1 and A2 were based on the regression model. For A3, confidence intervals were bootstrapped (10,000 iterations).
